# Coronary artery ectasia in a patient with myocardial infarction

**DOI:** 10.5830/CVJ-21.019

**Published:** 2011

**Authors:** Jesuraj ML, Mukerjee D, Singh R, Agarwal B, Jesuraj AV

**Affiliations:** Institute of Cardiovascular Sciences, IPGME&R, Kolkata, West Bengal, India; Institute of Cardiovascular Sciences, IPGME&R, Kolkata, West Bengal, India; Institute of Cardiovascular Sciences, IPGME&R, Kolkata, West Bengal, India; Institute of Cardiovascular Sciences, IPGME&R, Kolkata, West Bengal, India; Department of Pathology, VIMS, Kolkata, West Bengal, India

**Keywords:** coronary artery ectasia, myocardial infarction

## Abstract

We report on a case of triple-vessel coronary artery ectasia (CAE) in a young patient. This patient presented with anterior wall myocardial infarction (MI) with post-infarct angina. His coronary angiogram revealed coronary artery ectasia involving the left anterior descending, circumflex and right coronary arteries.

Coronary artery ectasia (CAE) is an uncommon disorder diagnosed in one to 4% of patients undergoing coronary arteriography. 1-3 CAE is usually considered a variant of atherosclerotic coronary artery disease.^1^-^3^ Coronary artery disease in young adults usually occurs in patients with multiple predisposing factors, such as hyperlipidaemia, cigarette smoking, diabetes mellitus, hypertension, and a strong family history. In the absence of predisposing factors, other causes should be considered, particularly mucocutaneous lymph node syndrome [Kawasaki disease (KD)].^4^

## Case report

A 36-year-old man presented with a history of acute anterior wall myocardial infarction (MI) with post-infarct angina. He was stabilised with medical therapy. There was no history of hypertension, diabetes mellitus or hyperlipidaemia. The patient was a non-smoker. He was unable to recall any definite symptoms of acute KD in childhood. The physical examination and laboratory data disclosed no abnormalities. The lipid profile, homocysteine and lipoprotein (a) [Lp(a)] levels were within normal limits.

**Fig. 1:**
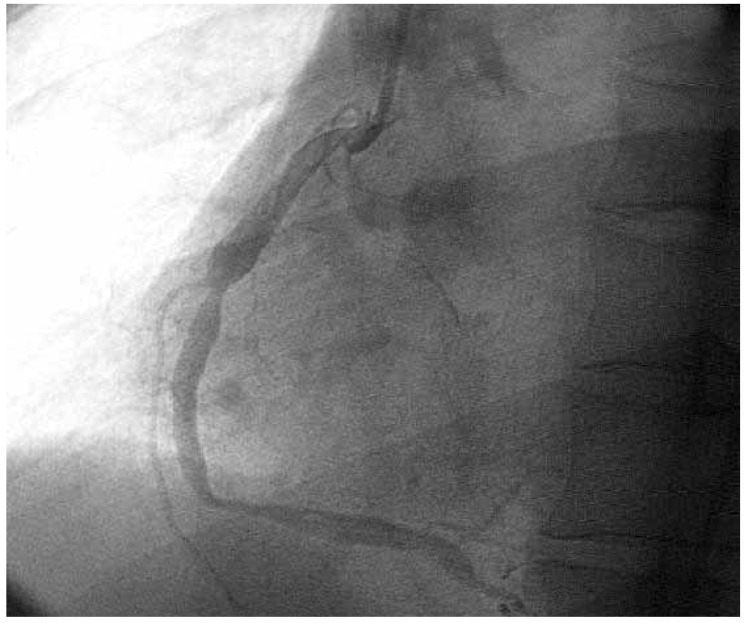
Selective left coronary angiogram demonstrating severe ectasia of the proximal segments of the left coronary artery with stenotic lesions.

**Fig. 2:**
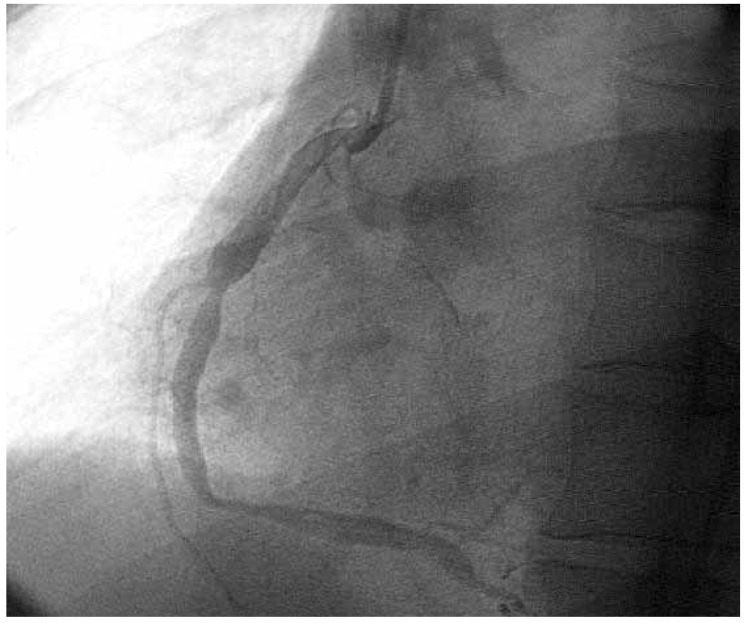
Selective right coronary angiogram showing multiple ectatic segments and stenosis of proximal and mid segments.

An ECG showed features of recent extensive anterior wall MI. A two-dimensional Doppler echocardiogram showed a left ventricular ejection fraction of 46% with anterolateral wall hypokinesis. Coronary arteriography (Figs 1, 2) demonstrated severe disease involving the left anterior descending, the circumflex and the right coronary arteries. The proximal segments of the arteries were very ectatic. Multiple aneurysms alternating with severe stenoses were seen along the entire length of the vessels, an appearance typically seen in KD.^4^ He was stabilised with medical therapy and referred for coronary artery bypass grafting.

## Discussion

KD is a febrile disorder of unknown cause with multiple systemic manifestations, affecting primarily infants and young children.^4^ First described in Japan by Kawasaki in 1967 as acute mucocutaneous lymph node syndrome, it has been reported with increasing frequency around the world. Involvement of the heart with acute myocarditis and coronary angiitis occurs in 25 to 50% of the patients during the acute phase, accounting for most of the mortality. However, 50% of the aneurysms regress spontaneously over a one- to two-year period, and therefore adult ischaemic heart disease secondary to KD is infrequent and occurs mostly in young adults.^4^

Obtaining a history of childhood Kawasaki disease is quite difficult because the diagnosis of acute KD is based on clinical criteria only, without specific laboratory testing, and therefore requires a high index of suspicion. In young adults, diagnosis is based on typical features in two-dimensional echocardiography and coronary arteriography. The former consists of local wall motion abnormalities as a result of prior MIs and ectasia or frank aneurysms of the proximal coronary arteries.^5^,^6^

Echocardiography is particularly helpful in the paediatric population, both for initial diagnosis and for long-term follow up.^5^,^6^ Coronary arteriography typically reveals multivessel aneurysmal disease alternating with segmental stenoses, coronary ectasia, calcifications, rich collateral circulation, and varying degrees of left ventricular dysfunction as a sequela of multiple MIs or myocarditis, or both.^4^,^6^

## Conclusion

We believe that our patient had KD rather than atherosclerotic CAE, for the following reasons. (1) He had extensive triplevessel disease at a very early age. He was a non-smoker without any other risk factors for atherosclerotic coronary artery disease (CAD). This would favour KD because patients with CAE have the typical risk profile of atherosclerotic CAD.^1^-^3^,^6^ (2) Our patient had ectasia involving all three vessels, which is rare in atherosclerotic CAE, but typical of KD.^1^-^4^ In our patient, echocardiography failed to reveal features of KD because the very proximal left main and right coronary arteries were spared. This is not uncommon in KD.^4^,^6^ However, coronary arteriography clearly demonstrated the typical findings of KD.
